# Supplementary material for: Analysis of PPARγ Signaling Activity in Psoriasis
Source: Int J Mol Sci. 2021 Aug 10;22(16):8603. doi: 10.3390/ijms22168603 (PMC8395241; doi:10.3390/ijms22168603)
Supplement: Supplementary file 1 [file ijms-22-08603-s001.zip › Supplemental materials_Analysis of PPARg signaling activity in psoriasis/Pathway models/Models images and html files/PPARG inhibits phenotypes assotiated with psoriasis/136572.html]

inflammatory bowel disease


# Disease inflammatory bowel disease

|  |  |
| --- | --- |
| URN | urn:agi-meshdis:Inflammatory%20Bowel%20Diseases |
| Total Entities | 12 |
| Connectivity | 5009 |
| Name | inflammatory bowel disease |

---

|  |  |
| --- | --- |
| ChildConcepts | Crohn's disease |
|  | ulcerative colitis |
|  | cutaneous photosensitivity-lethal colitis syndrome |
|  | chronic granulomatous disease |
|  | immune dysregulation-inflammatory bowel disease-arthritis-recurrent infections syndrome |
|  | immune dysregulation-inflammatory bowel disease-arthritis-recurrent infections-lymphopenia syndrome |
|  | inflammatory bowel disease-recurrent sinopulmonary infections syndrome |
|  | IL21-related infantile inflammatory bowel disease |
|  | neonatal inflammatory skin and bowel disease |
|  | experimental inflammatory bowel disease |
|  | undetermined colitis |
|  | cap polyposis |

---

|  |  |
| --- | --- |
| Pathway | Proteins Involved in Inflammatory Bowel Diseases |
|  | PPARG inhibits phenotypes assotiated with psoriasis |
|  | Neighbors of inflammatory bowel disease |

---

|  |  |
| --- | --- |
| MedScan ID | 9018560 |

---

|  |  |
| --- | --- |
| Alias | Inflammatory Bowel Disease |
|  | inflammatory diseases of the bowel |
|  | Inflammatory Bowel Diseases |
|  | inflammatory disease of the bowel |

---

|  |  |
| --- | --- |
| MeSH ID | D015212 |
|  | M0023374 |

---

|  |  |
| --- | --- |
| MeSH Heading | Inflammatory Bowel Diseases |

---
